# Cerebral hemodynamics and functional connectivity changes in stroke patients with dysphagia under acidic taste stimulation: a preliminary study

**DOI:** 10.3389/fneur.2025.1533099

**Published:** 2025-06-05

**Authors:** Jiliang Kang, Junyue Lu, Mengbi Gu, Shuang Gong, Xiaohan Li, Xiaojuan Li, Lifeng Tang, Yu Jin, Youliang Wen, Min Tang

**Affiliations:** ^1^Ningbo Rehabilitation Hospital, Ningbo, Zhejiang, China; ^2^School of Rehabilitation Medicine, Gannan Medical University, Ganzhou, Jiangxi, China

**Keywords:** stroke, dysphagia, acidic taste stimulation, fNIRS, cerebral hemodynamics, functional connectivity

## Abstract

**Background:**

Swallowing difficulties after a stroke are a common complication that significantly impact the quality of life of patients. The cortical activation patterns in patients with dysphagia following a stroke may be influenced by different taste stimuli, but the underlying neural mechanisms remain unclear.

**Objectives:**

The aim of this study was to investigate the changes in brain cortical hemodynamic signals and functional connectivity in stroke patients with dysphagia during acidic taste stimulation.

**Methods:**

We recruited 15 patients with first-time swallowing difficulties due to stroke (53% male; mean age 69 ± 9.43 years; duration 2.47 ± 1.31 months post-stroke, onset between 2 weeks and 6 months). A 41-channel functional near-infrared spectroscopy (fNIRS) was used to measure changes in concentrations of oxygenated hemoglobin (HbO_2_) and deoxygenated hemoglobin (HbR) during taste stimulation. A one-sample t-test was used for cohort analysis. A two-sample t-test was used to compare cortical activation differences between pure water and acidic stimuli. Additionally, relative changes in HbO_2_ concentration throughout the experiment were extracted for functional connectivity analysis. The Pearson correlation coefficients of HbO_2_ concentrations across channels were analyzed in the time series, followed by Fisher Z-transformation, which was defined as the functional connectivity strength between channels.

**Results:**

During acidic taste stimulation, significant activation of multiple cortical regions, including Dorsolateral Prefrontal Cortex (DLPFC), Supplementary Motor Cortex (PSMC), and Primary Somatosensory Cortex (PSC) was observed compared to the neutral water condition (*p* < 0.05). Functional connectivity analysis revealed that the average functional connectivity strength of the cortical network during acidic taste stimulation was significantly higher than during the neutral water condition (acidic taste: 0.337 ± 0.134; neutral water: 0.249 ± 0.142, *p* = 0.03).

**Conclusion:**

This study demonstrates that acidic taste stimulation can significantly activate multiple cortical regions in stroke patients with dysphagia and enhance the connectivity strength of brain functional networks, which may have a positive effect on swallowing function regulation. These findings provide a theoretical basis for future taste-based neurorehabilitation interventions and offer new insights into the treatment strategies for dysphagia after stroke.

## 1 Introduction

Stroke is one of the leading causes of adult disability and death worldwide. According to the World Health Organization, millions of new cases of stroke occur annually. Strokes can be classified into ischemic and hemorrhagic types, with ischemic strokes being the most common. Strokes not only severely impact patients’ physical health but also significantly affect their quality of life and impose a burden on families. Dysphagia is a common sequela in stroke patients, with an incidence rate of 50 to 80% (1). Dysphagia not only affects patients’ food intake but also can lead to complications such as malnutrition, dehydration, and aspiration pneumonia, further increasing patient suffering and medical costs (2, 3).

The mechanism of dysphagia is complex, involving various neurophysiological processes. With the development of imaging technology, many studies have found cortical activation in the brain during swallowing, such as the cerebellum, anterior cingulate cortex, somatosensory cortex, primary motor cortex, supplementary motor area, and Broca’s area (4–6). Stroke can damage the neural network controlling swallowing, including multiple regions such as the cerebral cortex, brainstem, and spinal cord, and dysphagia can also occur in patients with unilateral stroke (7). Particularly, functional impairment in areas such as the frontal cortex, sensorimotor cortex, and primary motor cortex often leads to a decrease in swallowing reflex and a decline in swallowing coordination ability (8). These neurophysiological mechanisms of damage have a significant impact on the patient’s recovery process. Therefore, finding effective treatment methods to improve swallowing function has become an important direction in post-stroke rehabilitation research.

In recent years, taste stimulation, as a non-invasive intervention, has gradually attracted the attention of researchers. The role of sour taste stimulation in saliva secretion and swallowing reflex has been preliminarily confirmed. Some studies have found that sour taste stimulation can enhance the swallowing reflex and promote the activation of related neural areas, thereby improving swallowing function (9). This discovery suggests that the brain’s control over swallowing through taste stimulation may provide a new therapeutic approach for the rehabilitation of post-stroke dysphagia. Additionally, sour taste stimulation has also been found to enhance the hemodynamic activity of related brain areas, which means that taste stimulation may improve swallowing function by affecting cerebral blood flow (10). Previous studies have observed the effects of sour stimulation on stroke patients with dysphagia, from airway damage grading to detailed kinematic and morphological data, sensory evaluation, electromyography, manometry, and various neuroimaging measurements (11, 12). However, the specific mechanisms of the effects of sour taste stimulation on stroke patients are still unclear, especially in terms of cerebral blood flow dynamics and functional connectivity, and research in these areas is still relatively scarce. Therefore, this study aims to explore the effects of sour taste stimulation on the cerebral blood flow dynamics and functional connectivity of stroke patients, providing a scientific basis for clinical rehabilitation.

Previous studies have used magnetic resonance imaging (MRI) to investigate the function of the cerebral cortex related to swallowing (13, 14), but its applicability to the assessment of swallowing function is low. Later, researchers used a neuroimaging instrument more suitable for the assessment of swallowing function, functional near-infrared spectroscopy (fNIRS). The fNIRS technique relies on specific laser wavelengths (usually in the range of 700–1,700 nm) penetrating through the scalp to enable non-invasive measurement of changes in brain activity (15). It is widely used in clinical settings due to its safety, low cost, portability, excellent temporal resolution (compared to functional magnetic resonance imaging), and moderate spatial resolution. fNIRS monitors the relative changes in the concentration of oxygenated hemoglobin (HbO_2_) and deoxygenated hemoglobin (HbR) in cerebral cortex blood flow using near-infrared light, thereby indirectly reflecting brain functional activity (16, 17). fNIRS studies investigating the hemodynamic response to taste stimulation are relatively rare, but previous fNIRS studies have successfully shown different changes in hemodynamic signals when healthy adults swallow different liquids [e.g., tasteless versus flavored broth (18), or sour water versus sweet water versus distilled water (10, 19)].

In summary, research on post-stroke dysphagia has important clinical value, especially in finding new rehabilitation strategies. Sour taste stimulation, as a potential intervention, requires further exploration of its mechanism of action on stroke patients. This study uses fNIRS technology to assess the changes in cerebral blood flow dynamics and functional connectivity of stroke patients under sour taste stimulation. We hope that this study will provide new theoretical support for the swallowing rehabilitation of stroke patients and may also open up new directions for future taste-based rehabilitation treatments.

## 2 Materials and methods

### 2.1 Participants

The study subjects were selected from stroke patients admitted to the Neurorehabilitation Department of Ningbo Rehabilitation Hospital from September 2023 to May 2024. All patients met the following inclusion criteria: (1) first-time unilateral stroke, diagnosed by computed tomography (CT) or magnetic resonance imaging (MRI); (2) patients with stable vital signs; (3) the course of the disease was between 2 weeks and 6 months, and the age was between 40 and 80 years old; (4) dysphagia determined by fiberoptic endoscopic evaluation of swallowing (FEES); (5) patients who could understand and cooperate with fNIRS assessment and voluntarily signed an informed consent form. Exclusion criteria are as follows: (1) patients with a history of stroke, cerebral hemorrhage, or other neurologic diseases; (2) patients with severe cognitive impairment or aphasia; (3) patients with taste disorders; (4) patients with a history of intracranial metal implants, skull defects, or other contraindications for fNIRS examination; (5) patients with a history of sedation, antidepressant medication; (6) patients with a history of medication that may change cortical excitability within 2 months. This study follows the Helsinki Declaration and relevant international guidelines. The Ethics Committee of Ningbo Rehabilitation Hospital (approval number 2023–24) granted ethical approval.

### 2.2 Activation task

**Figure 1:**
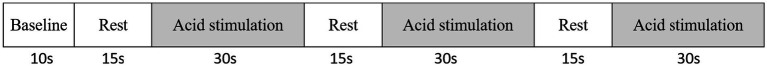
The experimental procedure.

The acidic taste stimulation condition was neutral acid (0.1 M citric acid) (10), and the tasteless condition used pure water (20), both solutions were at room temperature. The experimental paradigm was divided into a taste stimulation phase and a rest phase. Before the experiment, a cotton swab dipped in citric acid solution was applied to the subject’s mouth, and the subject was allowed to correctly perceive the sour taste before proceeding with the experiment. All participants were asked to remain seated in a relaxed position with their hands on their knees. After communicating the experimental process with the participants, no speaking was allowed. The experimental process included three stages, each consisting of a 15-s rest phase and a 30-s taste phase. During the taste stimulation phase, participants were asked to slightly open their mouths, and the experimenter used a cotton swab soaked in citric acid or pure water to stimulate the tip of the tongue, both sides of the tongue, the root of the tongue, and both sides of the oral cavity. During the rest period, participants were asked to remain relaxed and not swallow. The order of acid stimulation and pure water stimulation was randomized, and after each taste stimulation task, participants rested for 5 min. After the acid stimulation, participants were asked to rinse their mouths to avoid residual effects. The specific experimental process is shown in Figure 1.

### 2.3 Measurement of fNIRS

**Figure 2:**
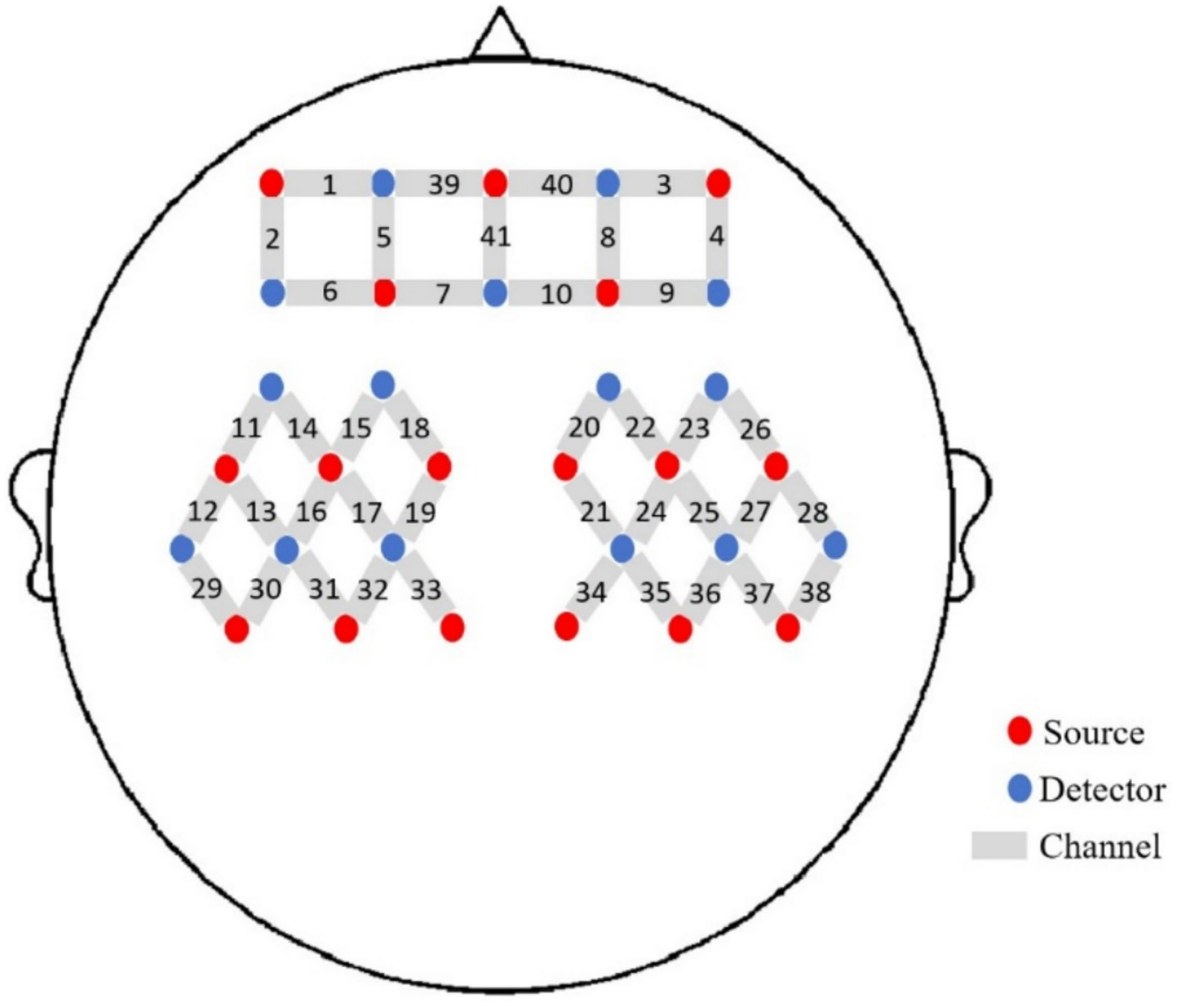
The placement of the probes.

We used a 41-channel fNIRS system (Danyang Huichuang Medical Equipment Co., Ltd., China) to measure the concentration changes of HbO_2_ and HbR under rest and taste stimulation at two wavelengths of 730 and 850 nm of infrared light, based on the modified Beer–Lambert law. With a sampling rate of 10 Hz, 16 light sources and 15 detectors were placed on the scalp of the subjects. The probe positioning is shown in Figure 2. To standardize the conditions and prevent environmental light from entering the system, each optical signal was attached to the skull surface with a custom hard plastic cap. The brain region for fNIRS recording was chosen according to the widely adopted 10/10 electrode system, with Cz positioned between optodes S6 and S16 (21). A 3D digitizing system was employed to obtain the MNI coordinates for each participant (22), and the corresponding brain area was identified based on the MNI and Talairach coordinates (23). The contribution of each Brodmann area in each channel was determined based on the MNI coordinate outputs.

### 2.4 Preprocessing and analyzing of fNIRS data

fNIRS data were analyzed using NirSpark (Danyang Huichuang Medical Equipment Co., Ltd., China). The following pre-processing was performed on the fNIRS data. First, interference cycles and pseudo-signals were eliminated, and light intensity was converted to optical density. Second, a Butterworth band-pass filter of 0.01–0.2 Hz was used to eliminate slow drift and cardiac pulsation. Third, optical density was converted to blood oxygen concentration. Finally, “0–45 s” was set as the block paradigm time for the hemodynamic response function (HRF), and “-2–0 s” was set to retain the baseline state. The blood oxygen concentrations of the three block paradigms were superimposed and averaged to obtain the block average results. For each pre-processed experimental data, a general linear model (GLM) was used to perform individual-level analysis for each channel. Next, the HbO_2_ concentration between the rest period and the taste stimulation period was compared using a single-sample t-test and significance threshold setting for each channel, and the false discovery rate (FDR) correction was performed. When the *p*-value of a channel is less than 0.05, it indicates that there is a significant difference in HbO_2_ concentration between the rest period and the taste stimulation period, suggesting that the cortical area of that channel is activated.

To explore the mechanism of post-stroke dysphagia, we used functional connectivity analysis to observe the whole-brain functional connectivity of post-stroke dysphagia patients during the taste stimulation task. In the network module of the NirSpark software package, the relative changes in HbO_2_ concentration during the entire experiment were extracted for functional connectivity analysis. The Pearson correlation coefficient of HbO_2_ concentration in each channel was analyzed on the time series. Then, Fisher *Z* transformation was performed, and the transformed values were defined as the strength of functional connectivity between channels. FDR-adjusted *p*-values are reported for all connectivity results.

## 3 Results

### 3.1 Study population

A total of 15 patients (8 males and 7 females) participated in our study, and they were all included in the analysis. Their average age was 69 ± 9.43 years, with a PAS score of 5.87 ± 1.20 points and an average disease course of 2.47 ± 1.31 months. The basic characteristics of each post-stroke dysphagia patient are shown in Table 1.

**Table 1:** Characteristics of patients with post-stroke dysphagia.

Patients	Age (years)	Gender	Duration after onset (months)	Stroke type	PAS score
1	80	Female	3	Ischemic	5
2	60	Female	5	Ischemic	5
3	62	Male	1	Ischemic	8
4	74	Male	1	Ischemic	5
5	80	Male	1	Hemorrhage	6
6	76	Male	4	Ischemic	7
7	79	Female	4	Ischemic	6
8	63	Male	1	Ischemic	6
9	50	Female	2	Hemorrhage	8
10	58	Male	4	Hemorrhage	4
11	72	Male	3	Ischemic	6
12	75	Male	1	Ischemic	4
13	69	Female	3	Hemorrhage	7
14	76	Female	2	Ischemic	6
15	53	Female	3	Ischemic	5
All	69 ± 9.43	–	2.47 ± 1.31	–	5.87 ± 1.20

### 3.2 Cortical activation under acidic taste stimulation

**Figure 3:**
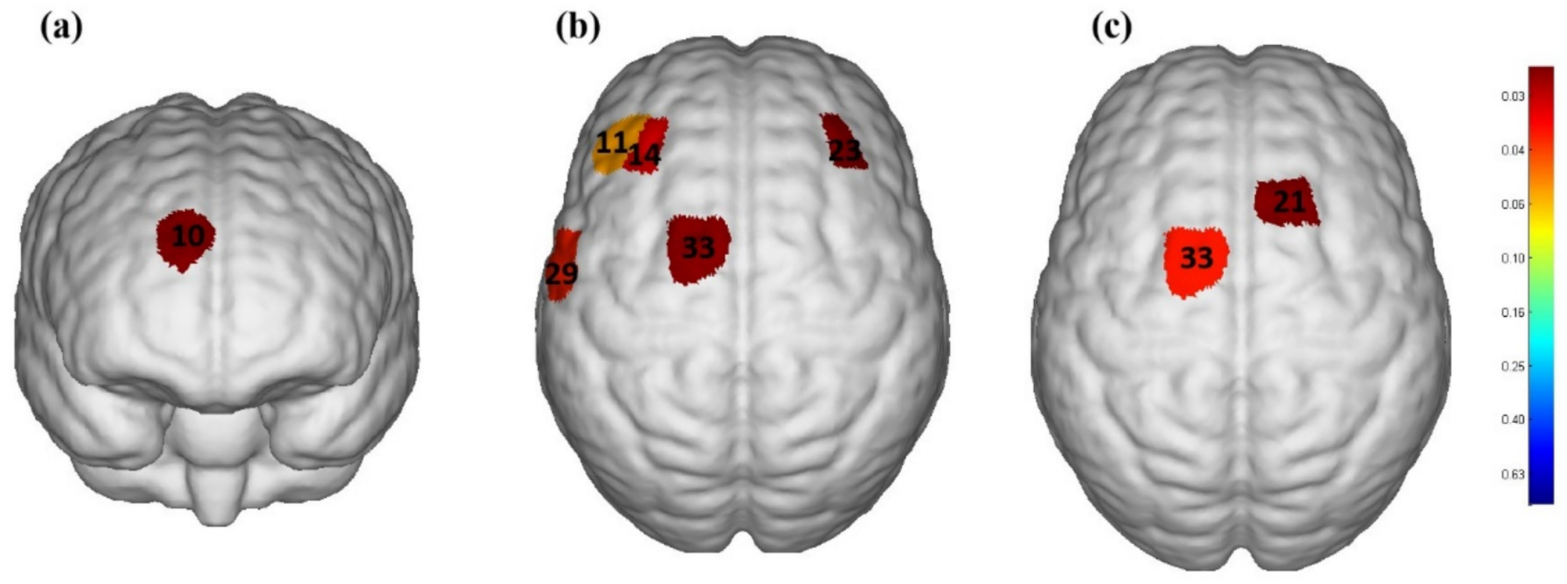
The cerebral cortex activation map during performance of stimulation task based on beta values (group-average map). **(a)** Significantly activated channels in the tasteless pure water stimulation group. **(b)** Significantly activated channels in the acidic taste stimulation group. **(c)** Comparison of brain activation levels between pure water stimulation and acid stimulation. Only the areas corresponding to the significantly activated channels were shown. The color gradient on the right represents the *p*-value. *p* < 0.05 for significant activation. The redder the color, the smaller the *p*-value.

Figure 3 shows the activation areas of the cerebral cortex in subjects during the taste stimulation task. Figure 3a shows that the activation area was quite limited under the tasteless pure water stimulation, with mild activation in the Dorsolateral Prefrontal Cortex (DLPFC) and Frontopolar area (*p* = 0.035). However, Figure 3b shows that under acidic taste stimulation, multiple areas of the cerebral cortex showed widespread activation, including the DLPFC (*p* = 0.01), Pre-Motor and Supplementary Motor Cortex (PSMC) (*p* = 0.003), and Primary Somatosensory Cortex (PSC) (*p* = 0.02). Figure 3c shows that compared with the non-taste condition (pure water), acidic taste stimulation significantly activated the PSMC area (*p* < 0.05). Supplementary Table 2 compares the activation of the cerebral cortex during the taste stimulation task between the tasteless condition group and the acid stimulation group based on the β values. The acid stimulation group had significantly higher β values in 2 channels than the tasteless condition group (*p* = 0.01, *p* = 0.03). The multi-channel β values for the two groups of subjects are shown in Supplementary Table 2.

### 3.3 Functional connectivity strength

**Figure 4:**
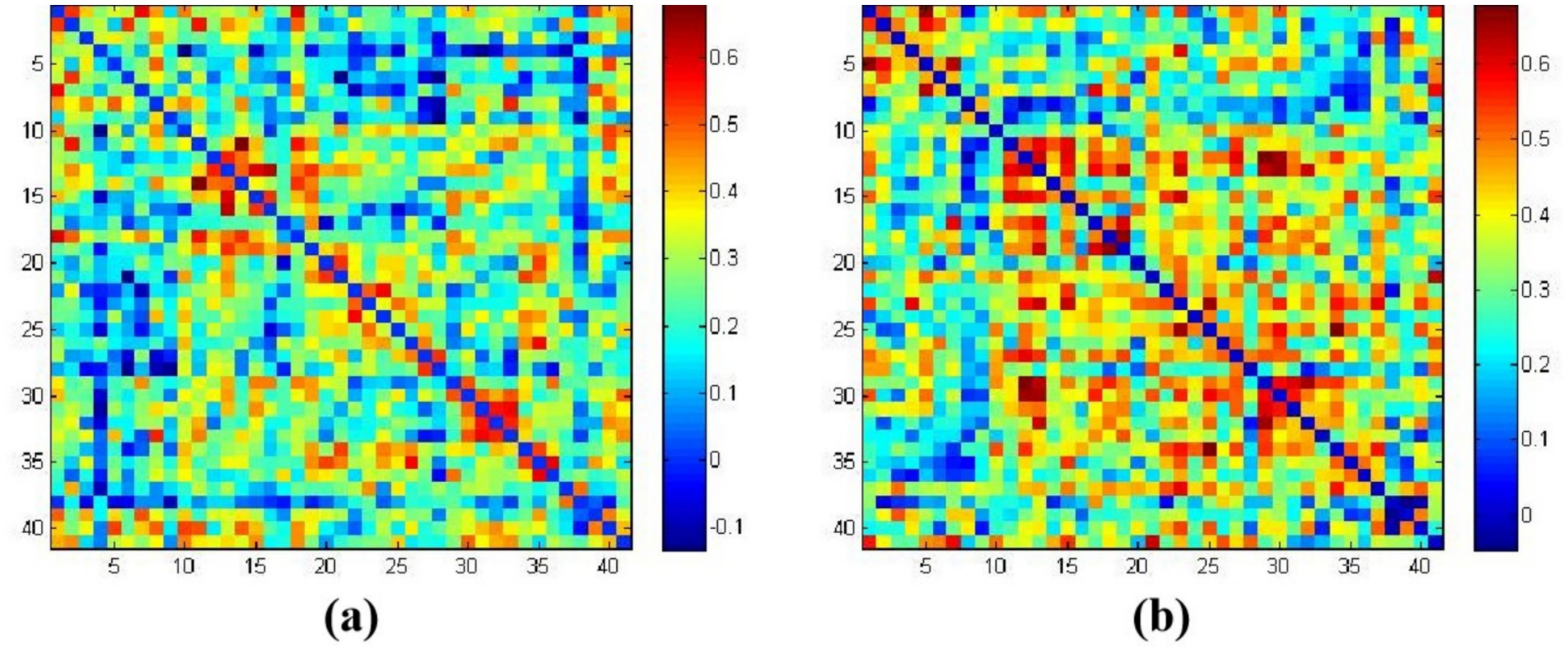
The average functional connectivity strength based on HbO_2_. **(a)** Correlation coefficients matrix between channels in the tasteless pure water stimulation group; **(b)** correlation coefficients matrix between channels in the acidic taste stimulation group. The color gradient on the right represents the beta values.

Figures 4a,b represent the average functional connectivity strength between cortical channels under tasteless stimulation (water) and acidic taste stimulation, respectively. The average brain functional network connectivity strength under acidic taste stimulation was higher than under tasteless. The average functional connectivity strength under tasteless stimulation (water) was 0.249 ± 0.142, and under acidic taste stimulation, it was 0.337 ± 0.134 (*p* = 0.03). Raw and FDR-corrected *p*-values in Supplementary Table 3.

## 4 Discussion

### 4.1 The impact of acidic taste stimulation on cortical activation in stroke patients

Taste information is initially formed in the taste buds on the tongue, travels through the afferent gustatory nerves to the sensory ganglion neurons, and finally reaches the multiple taste centers of the brain (Brainstem solitary nucleus) (24). Our aim was to observe the changes in hemodynamic signals and brain functional connectivity in stroke patients with dysphagia during acidic taste stimulation using fNIRS, and to further explore the mechanism of post-stroke dysphagia. This study found that acidic taste stimulation significantly activated multiple brain areas related to swallowing, especially the DLPFC, PSMC, and PSC. Previous studies have shown increased cerebral blood flow activity in S1, anterior cingulate cortex, insula, SMA, inferior frontal gyrus, and inferior parietal lobule during sour taste stimulation (25). This study also observed that compared with pure water stimulation, the PSMC area was significantly activated under acidic taste stimulation, while pure water stimulation activated a smaller area of the cortex, with only mild activation in the DLPFC and Frontopolar area. This finding is consistent with previous studies (26), indicating the potential importance of taste stimulation in the recovery of swallowing function. Sour taste requires higher sensory processing, swallowing reflex, and physiological response, involving the comprehensive action of brain areas including sensation, movement, and regulation (27). Pure water stimulation is milder and mainly activates the prefrontal areas related to cognitive control through a more basic sensory experience, so the activated area is smaller and the activation degree is lower (28). Sour taste may promote the initiation of swallowing reflex and saliva secretion, thereby improving swallowing ability (9). Understanding this mechanism provides a new therapeutic approach for clinics, indicating that sour taste stimulation may become an important auxiliary means for the rehabilitation of post-stroke dysphagia.

The study also showed that the connection strength between DLPFC and PSMC under sour taste stimulation was significantly enhanced. This enhanced connectivity not only reflects more effective information transfer and neural network collaboration but also indicates that in stroke patients, rehabilitation training for dysphagia can consider combining taste stimulation to promote the recovery of brain function. This non-invasive intervention method is safer and more acceptable compared to drug treatment and surgical methods, opening up a new direction for clinical application.

### 4.2 The significance of functional connectivity

The role of functional connectivity in neurorehabilitation is increasingly valued, and this study further explores the impact of sour taste stimulation on the brain functional connectivity of stroke patients. The results show that sour taste stimulation significantly enhances the connection strength between DLPFC and PSMC. This finding indicates that local brain damage in stroke patients may lead to a decrease in neural network connectivity, thereby affecting their coordination ability and efficiency during swallowing. Previous studies (29, 30) have explored the functional connectivity of cortical networks during swallowing tasks, but they did not observe post-stroke dysphagia patients. Choi and Pyun (31) and others have demonstrated through fMRI that the brains of patients with chronic dysphagia have functional connections, and dysphagia may be a series of neurobiological manifestations caused by changes in the functional connections of brain structures related to swallowing. Babaei et al. (32) found that some subjects showed a connection advantage in the right hemisphere of the brain, while others had higher functional connectivity in the left primary motor cortex. Wen et al. (33) observed the brain structural functional connections between post-stroke dysphagia patients and healthy subjects during voluntary swallowing through fNIRS and found that the average functional connectivity of the cortical network in healthy individuals was stronger than in stroke patients. No studies have yet revealed the cortical connection strength of stroke patients under sour taste stimulation, and our study fills this gap.

This research result has important clinical significance, indicating that in the rehabilitation process, we should not only focus on the clinical symptoms of patients but also pay attention to the overall functional state of the brain network. Enhancing these connections, especially the connection between DLPFC and PSMC, may be an important way to improve swallowing function. In addition, the sour taste stimulation mentioned in the study, as a non-invasive intervention method, provides new ideas for rehabilitation treatment. The application of this method can activate related brain areas, promote neural plasticity, and help patients recover damaged neural functions.

In future studies, more systematic and comprehensive rehabilitation strategies can be explored, combining various intervention methods such as exercise training, cognitive training, and taste stimulation to enhance the functional connectivity of patients’ brains. Through interdisciplinary cooperation, researchers can design more effective rehabilitation plans, not only focusing on a single treatment method but through multi-dimensional interventions to improve the overall rehabilitation effects of patients. For example, combining traditional physical therapy with new neurofeedback training, using modern technology to monitor patients’ brain activity, and providing data support for the formulation of personalized rehabilitation plans.

### 4.3 Clinical application potential of sour taste stimulation

Considering the results of this study, sour taste stimulation, as a potential treatment method, has the possibility of being widely applied in the rehabilitation of stroke patients. By increasing sour foods in patients’ daily diets or using specially designed sour taste stimulation tools, a simple and feasible rehabilitation method can be provided for patients (34). This method not only aims to improve patients’ swallowing function but also may significantly improve patients’ overall quality of life by enhancing the dining experience (35). Studies have shown that appropriate sour taste stimulation can activate saliva secretion, promote appetite, help patients adapt to diets better, and reduce the risk of malnutrition due to swallowing difficulties (36).

However, when applying sour taste stimulation in clinical practice, it is necessary to be cautious about the individual differences among different patients. Some patients may have significant differences in the perception and acceptance of sour taste, and even have different preferences or discomfort reactions to sour taste (37). Therefore, in the implementation process, individualized adjustments should be made according to the specific situation of each patient to ensure its effectiveness and safety. This includes assessing patients’ dietary habits, taste preferences, and physical reactions to formulate a sour taste stimulation plan suitable for their personal needs. In addition, the medical team should regularly monitor patients’ reactions and adjust the intensity and method of stimulation in a timely manner to achieve the best rehabilitation effect. Through this comprehensive personalized strategy, the medical team can not only improve patients’ swallowing ability but also effectively improve patients’ overall treatment experience, providing more comprehensive support for the patient’s rehabilitation journey.

## 5 Study limitations

Although this study provides important preliminary evidence, it also has some limitations. Firstly, the sample size is relatively small, with only 15 stroke patients, which may limit the statistical power and generalizability of the study results. Future research should expand the sample size to improve the generalizability and reliability of the findings, and conduct multiple independent repeated experiments to ensure the robustness and consistency of the results, thereby enhancing the statistical power. Second, this study only explored the effects of sour taste stimulation, and future studies can further explore the impact of other taste stimulations (such as sweet, bitter, salty, etc.) on stroke patients with dysphagia to determine the most effective taste stimulation method.

In addition, this study did not delve into the specific neural mechanisms. How taste stimulation affects neural plasticity, neural conduction, and information processing in the brain still needs further research. Understanding these mechanisms will not only help optimize treatment plans but also provide a foundation for future related research.

## 6 Conclusion

This study provides preliminary evidence that sour taste stimulation can significantly activate brain areas related to swallowing and enhance their functional connectivity. This finding provides new ideas for the rehabilitation of stroke patients, suggesting that taste stimulation as a potential non-invasive intervention method is worth further exploration and application in clinical practice. Future studies should further explore these mechanisms to optimize treatment plans for dysphagia and improve patients’ quality of life.

## Data Availability

The datasets presented in this study can be found in online repositories. The names of the repository/repositories and accession number(s) can be found in the article/Supplementary material.
